# Forebrain microglia from wild-type but not adult 5xFAD mice prevent amyloid-β plaque formation in organotypic hippocampal slice cultures

**DOI:** 10.1038/srep14624

**Published:** 2015-09-29

**Authors:** Sabine Hellwig, Annette Masuch, Sigrun Nestel, Natalie Katzmarski, Melanie Meyer-Luehmann, Knut Biber

**Affiliations:** 1 Department of Psychiatry and Psychotherapy, Freiburg, Germany; 2 Department of Neuroanatomy, Freiburg, Germany; 3 Department of Neurology, University of Freiburg, Freiburg, Germany; 4 Department of Neuroscience, University Medical Center Groningen, University of Groningen, Groningen, The Netherlands

## Abstract

The role of microglia in amyloid-β (Aβ) deposition is controversial. In the present study, an organotypic hippocampal slice culture (OHSC) system with an *in vivo*-like microglial-neuronal environment was used to investigate the potential contribution of microglia to Aβ plaque formation. We found that microglia ingested Aβ, thereby preventing plaque formation in OHSCs. Conversely, Aβ deposits formed rapidly in microglia-free wild-type slices. The capacity to prevent Aβ plaque formation was absent in forebrain microglia from young adult but not juvenile 5xFamilial Alzheimer’s disease (FAD) mice. Since no loss of Aβ clearance capacity was observed in both wild-type and cerebellar microglia from 5xFAD animals, the high Aβ_1−42_ burden in the forebrain of 5xFAD animals likely underlies the exhaustion of microglial Aβ clearance capacity. These data may therefore explain why Aβ plaque formation has never been described in wild-type mice, and point to a beneficial role of microglia in AD pathology. We also describe a new method to study Aβ plaque formation in a cell culture setting.

Extracellular deposition of amyloid-β (Aβ) in plaque form is one of the neuropathological hallmarks of Alzheimer´s disease (AD)^1^. The amyloid cascade hypothesis, which postulates that the deposition of the Aβ-peptide in the brain is a key event in AD pathology, has influenced AD research for several decades. However, a number of studies on therapeutics (i.e., anti-amyloid antibody treatment) intended to reduce Aβ production or aggregation have failed at various stages of development^2^, possibly because the mechanisms of Aβ plaque formation and maintenance remain poorly understood. Thus, a better understanding of this pathophysiological phenomenon may promote research into new treatment options for AD.

Microglia are the resident brain phagocytes whose role in Aβ plaque formation is debated^3^,^4^. On one hand, previous studies have shown that microglia are able to take up Aβ *in vitro*^5^,^6^,^7^,^8^. On the other hand, microglial depletion *in vivo* does not affect Aβ plaque load, as demonstrated in two AD mouse models in which the amyloid precursor protein (APP) was overexpressed^9^. Two aspects may explain the difference in microglia Aβ uptake capacity in *in vitro* vs. *in vivo* conditions. Recent evidence has suggested that cultured microglia display mRNA expression profiles that more closely resemble those of peritoneal macrophages, making them poor models for *in vivo* microglia^10^,^11^,^12^,^13^,^14^. Furthermore, it was found that *in vivo* depletion of microglia is followed by rapid repopulation of microglia-like cells of unknown origin, making it difficult to establish permanent microglia-free conditions *in vivo*^15^,^16^. Thus, a comprehensive analysis of the role of microglia in Aβ plaque development requires an experimental system that allows reliable microglia depletion *in vivo*.

Organotypic hippocampal slice cultures (OHSCs) serve as a powerful *in vitro* tool for studying cellular functions, since they maintain many structural and functional properties of the hippocampus *in vivo*. For example, OHSCs keep their *in vivo* capacity for supporting different types of neurons and glia, and also retain the complex three-dimensional (3D) organization of the hippocampus. Given that the hippocampus is a strategic region for memory encoding and exhibits early neurodegeneration in AD^17^,^18^, OHSCs have been used to study various aspects of AD pathology such as tangle formation, or neuronal loss as a marker of neurodegeneration^19^,^20^,^21^. Of note, Aβ plaque formation has hitherto not been observed or induced in OHSCs of wild-type mice. Thus far, transgenic mouse models of AD have been required to study cerebral Aβ plaque formation (for review see:^22^), as these do not form in wild-type mice and also cannot be induced^23^,^24^,^25^. However, the mechanistic basis for the lack of Aβ plaque formation in wild-type brain tissue has remained obscure.

Remarkably, it is possible to deplete and replenish microglia in OHSCs^26^,^27^. Importantly, we have shown that after replenishment, microglia rapidly acquire an *in vivo*-like distribution and the typical ramified morphology^26^. Moreover, microglia precursor cells differentiate into ramified microglia when applied to microglia-free OHSC^28^. Taken together, our previous findings indicate that OHSCs provide an appropriate cellular environment which allows the investigation of microglia in an *in vivo-like* setting, making them a suitable model to explore the role of microglia in Aβ plaque formation.

Against this background, the aims of the present study were two-fold:

1. To investigate the effect of microglia on cerebral Aβ plaque formation in OHSCs derived from wild-type mice.
2. To evaluate potential differences in amyloid-clearance capacity between microglia from wild-type and 5xFamilial Alzheimer’s disease (FAD) mice.

## Results

### Depletion of microglia induces the formation of amyloid-beta deposits in wild-type organotypic hippocampal slice cultures

**Figure 1:**
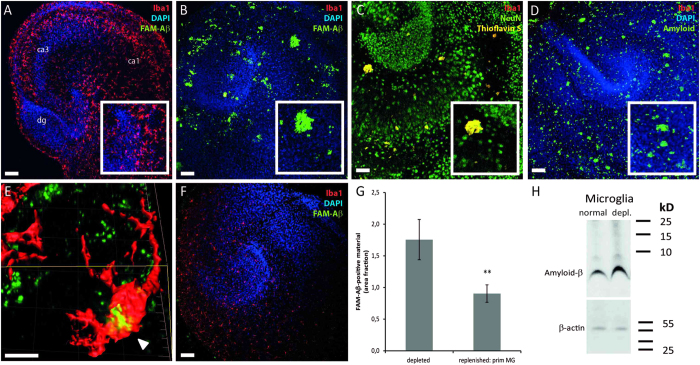
Microglia incorporate synthetic amyloid-β and prevent plaque formation. (**A**,**B**,**D**,**E**) Iba1/DAPI (red/blue) staining on wild-type OHSC (dg = dentate gyrus; ca1/ca3 = cornu ammonis 1/3) following treatment with FAM-labeled Aβ_1−42_ indicates a lack of plaque formation in microglia-containing slices (**A**). In contrast, microglia depletion resulted in an increase of Aβ plaques (**B**). Higher magnification reveals plaque-like structures (insert in (**B**)). Thioflavin S staining (**C**) and Aβ immunohistochemistry (**D**) confirmed the formation of Aβ plaques. (**E**) Confocal analyses of OHSC demonstrate the microglial uptake of Aβ (arrowhead). (**F**) Replenishment of microglia-depleted OHSC with primary forebrain microglia significantly reduces the Aβ plaque-load. Similar results were obtained in at least 4 independent experiments (**G)** Quantification of Aβ positive material using Image J revealed reduced Aβ-positive area fraction (0.903 ± 0.138%) in replenished slices compared to microglia-free slices (1.756 ± 0.318%) (Data are expressed as mean ± SEM from n = 2 experiments with 11 slices/group; Mann-Whitney *U*; *p = 0.038). (**H**) Quantitative Western immunoblot analysis of Aβ fragments using 6E10 antibody and densitometric analyses revealed significantly higher Aβ levels in microglia-free slices (189.31 ± 7.39%) compared to microglia-containing tissue (100.00 ± 14.11%; Student’s *t* test; *p* = 0.001). Scale bars: A, B, C, D, F: 100 μm; E: 10 μm.

To elucidate the potential influence of microglia on Aβ plaque formation, synthetic 5-carboxyfluorescein (5-FAM)-labeled human Aβ_1−42_ was first applied to OHSCs, and slices were examined two weeks later. As expected, treatment of microglia-containing wild-type OHSCs with synthetic 5-FAM-labeled Aβ_1−42_ (n = 4; each 2 μl of 15 μM Aβ) did not result in plaque formation (Fig. 1A). In contrast, in microglia-free slices numerous plaque-like structures (green fluorescence) appeared within 14 days (Fig. 1B). To verify the existence of plaque-like structures and rule out artifacts of 5-FAM labeling, unlabeled synthetic Aβ_1−42_ was administered. As an indicator of Aβ plaque formation either Thioflavin S staining (yellow fluorescence) (Fig. 1C) or Aβ immunohistochemistry using 6E10 antibody (green fluorescence) (Fig. 1D) were used. Both staining procedures confirmed the occurrence of plaque-like structures only in the absence of microglia (Fig. 1C,D). Quantitative western blotting analyses revealed significantly higher levels of Aβ protein in microglia-free slices (189.31 ± 7.39% vs. 100.00 ± 14.11%; n = 4 experiments; Student’s *t* test; *p* = 0.001) (Fig. 1H). On a single cell level, confocal imaging and three-dimensional (3D) surface reconstruction of microglia showed intracellular Aβ material (6E10 staining, green fluorescence and Iba1 staining, red fluorescence) (Fig. 1E). Furthermore, replenishment of microglia-free slices with cultured primary microglia significantly abolished Aβ plaque formation (Fig. 1G,F). Taken together, our data indicate that Aβ plaques do not form in OHSC, while microglia are present.

### Microglia ingest amyloid-beta to the lysosomal compartment

**Figure 2:**
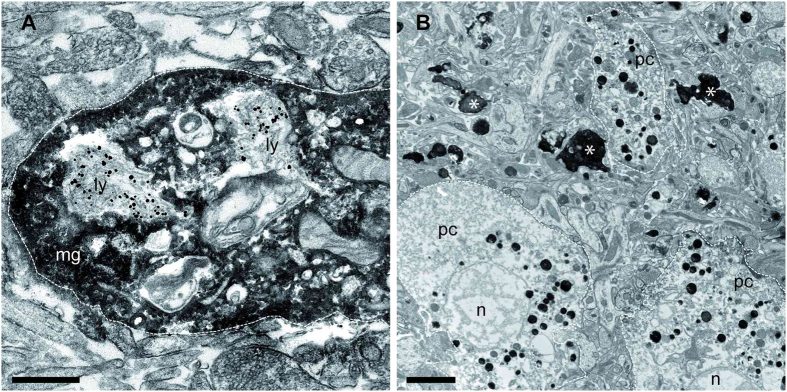
Ultrastructural analyses reveals ingestion of amyloid-β to the lysosomal compartment of microglia. (**A**,**B**) Immuno-electron microcroscopy using Iba1-DAB-staining as a microglial marker and immunogold-labeling for 6E10 in wild-type OHSC following treatment with synthetic amyloid-β (Aβ). (**A**) A high magnification electron micrograph revealed incorporation of Aβ into the lysosomal compartment (ly) of microglia (mg; white line) (**B**). In the absence of microglia Aβ is detected in pyramidal cells (pc, white line) showing signs of nuclear degeneration (n, nucleus) and in forms of extracellular plaques (asterisks). Scale bars: A: 2,500 nm B: 500 nm.

Next, an ultrastructural analysis with immuno-electron microscopy was performed to substantiate the confocal microscopy findings on Aβ plaque formation in wild-type OHSCs. Furthermore, we aimed for a more detailed morphological characterization of Aβ positive structures. 6E10-labeled Aβ (silver-intensified immunogold labelling) was observed in the lysosomal compartment of Iba1-positive microglia (DAB staining) (Fig. 2A). These findings suggest that aside from incorporating amyloid, microglia diminish Aβ load by digesting it. In OHSCs depleted from microglia, Aβ was detected both extracellularly in plaques and intracellularly in neurons, with hallmark signs of degeneration such as nuclear fragmentation and loss of cellular membrane integrity (Fig. 2B). The latter two were never observed in microglia-containing slices. Our data are therefore in line with earlier findings indicating that intraneuronal Aβ accumulation causes neurodegeneration^29^.

### The capacity to incorporate Aβ is absent in forebrain microglia from young adult but not juvenile 5x Familial Alzheimer’s disease mice

**Figure 3:**
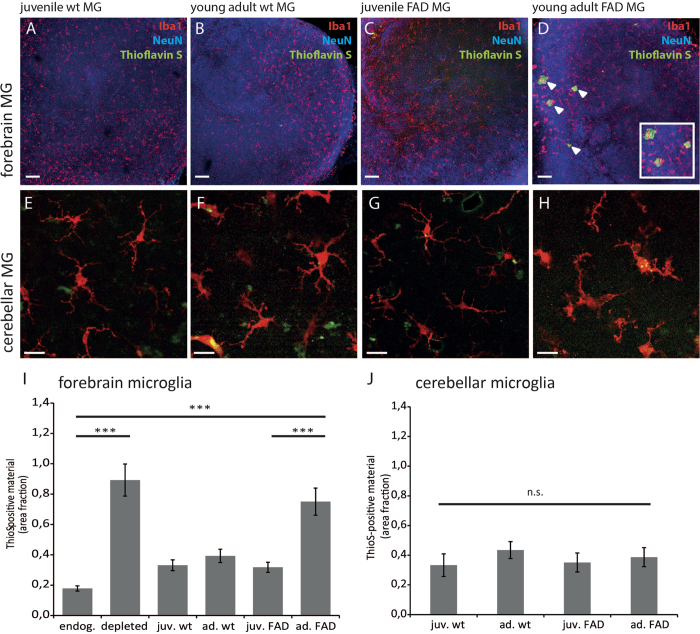
Forebrain microglia from adult FAD mice do not prevent Aβ-plaque formation. **Upper Panel** Low magnification images of microglia-depleted wild-type OHSC following replenishment with either 5-weeks-old (**A**,**C**) or 6-months-old forebrain microglia (**B**,**D**) from wild-type (**A**,**B**) or 5xFAD mice (**C**,**D**). Replenished microglia (Iba1/red) were evenly distributed throughout the slice (neuronal staining by NeuN/blue). Thioflavin S staining (green) revealed plaque-like structures ((**D**) insert) only in OHSCs replenished with microglia from adult 5xFAD mice. Scale bar: 100 μm **Lower Panel** High magnification images of replenished cerebellar microglia (Iba1/red) from either 5-weeks-old (**E**,**G**) or 6-months-old (**F**,**H**) wild-type (**E**,**F**) or 5xFAD mice (**G**,**H**). Replenished microglia (Iba1/red) were evenly distributed throughout the slice and acquired a ramified morphology (**E**–**H**). Scale bar: 10 μm. Densitometric analysis of ThioflavinS-positive material in OHSCs was performed by Image J. ANOVA followed by Scheffé post-hoc test revealed that wild-type juvenile (0.331 ± 0.036%, n = 35, p < 0.001) or adult forebrain microglia (0.393 ± 0.043%, n = 33, p < 0.001) impeded plaque formation compared to endogenous wild-type microglia (0.178 ± 0.017%, n = 33, p < 0.001). No significant difference was found between microglia-free OHSCs (0.892 ± 0.106%, n = 30) and slices replenished with forebrain microglia from adult 5xFAD mice (0.750 ± 0.089%, n = 20), while forebrain microglia from juvenile 5xFAD mice prevented plaque formation (0.318 ± 0.033%, n = 31, p < 0.001) compared to endogenous wild-type microglia. Data are expressed as mean ± SEM from three independent experiments (**I**). In contrast one way ANOVA revealed no differences in densitometric analysis of ThioflavinS-positive material between microglia cells isolated from the cerebellum of juvenile or young adult wild type or 5xFAD mice. Number of OHSC analysed: juv.wt n = 18, ad.wt n = 11, juv.FAD n = 14, ad.FAD n = 16, data are expressed as mean ± SEM from two independent experiments (**J**).

Our data point towards a marked inhibitory influence of microglia on Aβ plaque formation. Drawing on findings which suggested no impact of microglia on Aβ plaque formation in transgenic mouse models of AD^9^, we next compared microglia from wild-type mice with those derived from 5xFAD animals. Microglia-depleted OHSCs were replenished with microglia from the forebrain (cortex and hippocampus) from either wild-type or 5xFAD mice. Furthermore, forebrain microglia from juvenile (5-week-old) and adult (6-month-old) mice were compared between genotypes. An initial in-depth morphological analysis by 3D single-cell reconstruction revealed that replenished microglia displayed no significant differences with respect to process length or the number of branch points (Suppl. Fig. 1A–E) and were evenly distributed within slices (Fig. 3A–D). Thus, irrespective of age or genotype, replenished microglia acquired an *in vivo*-like, ramifiedmorphology. Following replenishment with either 5-week-old (Fig. 3A) or 6-month-old wild-type microglia (Fig. 3B) and subsequent treatment with synthetic Aβ_1−42_, no Aβ plaque formation was detected in slices by Thioflavin-S staining. Similarly, slices replenished with microglia from 5-week-old 5xFAD animals displayed no plaque-like structures after Thioflavin-S staining (Fig. 3C). However, when slices were replenished with microglia isolated from 6-month-old 5xFAD mice, Aβ plaque formation was observed (Fig. 3D). Quantification of Thioflavin-S staining showed that Aβ plaque formation in slices replenished with microglia from 6-month-old 5xFAD mice occurred to a similar extent to that in microglia-free slices (Fig. 3I). All other microglia-containing slices did not display significant differences with respect to Aβ plaque formation (Fig. 3I), indicating that microglia from 6-month-old 5xFAD mice lost their capacity to prevent plaque formation.

To address whether this loss of function was due to the (i) age or genetic background of 5xFAD mice or (ii) to the presence of high Aβ levels or plaque load in the forebrain a similar experiment was performed using microglia from the cerebellum. The cerebellum in APP transgenic mice stays free of Aβ plaques due to low expression of the transgenes in that area^30^,^31^,^32^, thus microglia in the cerebellum reside in an Aβ-free brain area. When transferred into OHSCs also cerebellar microglia invaded the slices and acquired a ramified morphology comparable to their counterparts from the forebrain (Fig. 3E–H). After treatment with synthetic Aβ_1−42_ and quantification of Thioflavin S staining it was found that cerebellar microglia prevented plaque formation irrespective of the genetic background or the age of the animals (Fig. 3J).

## Discussion

We provide first evidence for Aβ plaque formation in OHSC derived from wild-type mice. Previous attempts using intracranial injections of distinct Aβ species resulted in Aβ plaque formation in transgenic mouse models of AD, but not in wild-type animals^23^,^24^,^25^. Likewise, in OHSC of wild-type mice Aβ deposits have never been described, though a successful modeling of tau-pathology and associated neurodegeneration was accomplished in this culture system^19^,^20^,^21^. Induction of plaque formation in wild-type animals in general failed for as yet unknown reasons. Numerous studies have investigated the role of microglia in the pathogenesis of AD; however, this remains a contentious issue. Our data suggest that the previously observed lack of Aβ plaque formation in wild-type mice relates to the Aβ clearance capacity of microglia^19^,^20^,^21^, since Aβ deposits exclusively formed in wild-type tissue when microglia had been depleted. This finding is corroborated by the observation that microglia take up and possibly digest Aβ in the lysosomal compartment thereby preventing plaque formation in OHSC. This is in line with earlier studies linking dysfunctional intracellular degradation mechanisms in the autophagy-lysosomal system with AD pathogenesis^33^,^34^. Pickford *et al.* showed that levels of beclin 1, which modulates APP metabolism and promotes neurodegeneration, were diminished in affected brain regions of patients with early AD. Microglial beclin 1 has been reported to regulate phagocytosis and is impaired in AD^35^. Moreover, we found that only in the absence of microglia intraneuronal Aβ deposits occur, which was associated with neuronal degeneration. Taken together, our data underline the proposed beneficial role of microglia by promoting phagocytosis, degrading and ultimately clearing Aβ, one of the pathogenic proteins deposited in AD^36^.

While microglia derived from 5-week-old 5xFAD mice were able to impede Aβ plaque formation *in vitro*, its counterparts isolated from Aβ depositing 6-month-old 5xFAD mice sustain a significant loss of this function. This is in good agreement with *in vivo* observations in this particular mouse model. These transgenic mice express the mutated human amyloid precursor protein as well as the mutated human PSEN-1 gene, which leads to a tremendous Aβ_1−42_ burden. Accordingly, the first signs of plaque formation are already detectable at 2 months of age^32^. In this light, our data may indicate that microglia from young mice in general are initially capable of preventing plaque formation, whereby a loss of this particular function is critical to Aβ plaque formation in 5xFAD mice. It could therefore be hypothesized that chronic exposure to high Aβ_1−42_ burden plays a causative role in the early exhaustion of microglial Aβ clearance capacity, which, in turn, potentially leads to premature senescence of the brain’s protective system^11^,^13^. In order to test this hypothesis we have replenished microglia-depleted OHSCs with microglia from the cerebellum, an Aβ free brain area. Thus, microglia in the cerebellum are not exposed to high Aβ_1−42_ burden. Different from the effects seen with forebrain microglia, no differences in Aβ plaque formation were observed when cerebellar microglia were present in the slices, i.e., Aβ plaques generally did not form. These data strongly support the notion that it is indeed both the chronic exposure to high Aβ_1−42_ levels and plaque burden in the forebrain that are causing an exhaustion of microglial Aβ clearance capacity. In contrast, these findings do not suggest that a general effect of aging or the genetic background of 5xFAD animals underlie the impairment of this particular microglia function.

Our data are in good agreement with recent findings, as it was described that microglia isolated from two different AD mouse models (APPPS1 and APP23) showed impaired phagocytic capacity and Aβ uptake which correlated to the Aβ plaque burden of the tissue from which the microglia were isolated^37^,^38^. It has furthermore been described that microglia of AD brains resemble aged microglia and show age-associated microglial dysfunction of phagocytosis, motility and morphology (reviewed in^39^). This loss of microglia function could explain why the deletion of microglia in adult AD mouse models did not affect Aβ plaque load^9^. The mechanisms by which elevated Aβ_1−42_ levels and plaque burden lead to the observed loss in microglia function remain to be established. Of particular relevance to our present findings on forebrain microglial function is the question of whether changes in CD33 expression, an immunoglobulin-like lectin and risk gene for AD, inhibit the clearance of Aβ in microglial cell cultures^7^. It has moreover become increasingly clear that neurotransmitters may also modulate microglia Aβ uptake^40^. Since OHSCs lack the input from other brain regions such as the prefrontal cortex or brain stem nuclei, the lack of this input may also affect the development of Aβ plaques in OHSCs. Forthcoming studies should therefore also address the potential influence of aminergic or glutamatergic neurotransmitters on Aβ plaque formation in OHSCs.

## Conclusion

In summary, this study reinforces the notion that the innate immune cells of the brain serve as key players in AD pathology. By taking advantage of the fact that the depletion and replenishment of microglia allows for the rapid assembly of chimeric OHSCs, we now describe a powerful experimental model to in which to study the role of microglia in Aβ plaque formation and maintenance.

## Methods

### Animals

5xFamilial Alzheimer’s disease (5xFAD) mice strain Tg6799^32^ were a kind gift from Marco Prinz, University Medical Center Freiburg. The 5xFAD transgenic mice have the following five mutations: Swedish (K670N and M671L), Florida (I716V) and London (V717I) in human APP695 and human PS1 cDNA (M146L and L286V) under the transcriptional control of the neuron-specific *Thy-1* promoter. Animals were bred for heterozygosity. As wild type controls FAD-negative littermates were used. Both male and female have been used in this study. Mice were bred under pathogen-free conditions in a temperature and humidity controlled vivarium with a 12 h light-dark cycle, food and water were available ad libitum. All animal experiments have been approved by and were performed in accordance with the guidelines of the Regierungspräsidium Freiburg legislation for animal experiments.

### Organotypic hippocampal slice cultures

Organotypic hippocampal slice cultures (OHSC) have been prepared from newborn (P0-P3) C57BL/6N mice and were cultured according to the interface method^26^. OHSC were kept for 7 days *in vitro* (div) at 35 °C in a humidified atmosphere (5% CO_2_) before treatment. Medium was changed every other day.

#### Microglia depletion and replenishment

Microglia were depleted specifically from freshly prepared slice cultures using the macrophage toxin clodronate (Merck-Millipore, cat. no. 233183). Clodronate disodium-salt was solved in ultra-pure H_2_O (Biochrom) with a concentration of 1 mg/mL. Freshly prepared OHSC were incubated with 100 μg clodronate per mL standard culture medium for 24 hours at 35 °C. Subsequently, OHSC were rinsed with warm phosphate buffered saline (PBS) carefully and placed on fresh culture medium. Microglia-depleted OHSC were kept at least for 7 div before experiment. Medium was changed every other day. Primary mixed glia cultures were prepared from newborn C57BL/6 mice as previously described in detail^41^. After two weeks in culture microglia were harvested by shake-off and used immediately to replenish microglia-free OHSC as described below. Juvenile or adult microglia were acutely isolated from forebrain (cortex/hippocampus) or cerebellum of 5-weeks- and 25-weeks-old 5xFAD mice or wild type control-littermates as previously described^42^. In brief, the tissue was dissociated mechanically to a single cell suspension and the microglia cells were separated by density gradient centrifugation^43^. All steps were carried out on 4 °C to inhibit a change in microglia activity status. After isolation adult microglia were used immediately to replenish OHSC. To do so, the cell number of isolated microglia was determined, cells were pelleted by centrifugation at 200 *g* for 10 min at 4 °C, and carefully re-suspended in culture medium to a final density of 1000 cells per μL. 2000 cells in 2 μL medium were added an each microglia-free OHSC. Cells were allowed to distribute and ramify for 12 to 14 days before further treatment.

#### Aβ_1−42_ treatment

Synthetic human amyloid β (Aβ) peptide comprising amino acids 1−42 either unlabeled or labeled with 5-carboxyfluorescein (5-FAM) was purchased from AnaSpec (cat. no. 24224 or 23525-05, respectively). The peptide was reconstituted in Dulbecco’s Phosphate-Buffered Saline (PBS) to a 15 μM solution. The solution was stored for 7 days at 37 °C and was mixed every day for 5 minutes by vortex. Aliquots were stored at −20 °C. Before usage on OHSCs, Aβ was sonicated for 10 min in an ultrasound water bath followed by 2 min vortex to break down potential aggregates. 2 μL of Aβ were dropped on top of each OHSC 4 hours after medium change. Treatment was repeated 4 times every other day, thus each slice was in total treated with 8 μL of a 15 μM Aβ solution.

To analyze the Aβ solution d*ot blot assay* was performed: 2 μl the labeled or unlabeled solution was transferred onto a nitrocellulose membrane (0.1 μm pore size; Whatman). Membranes were allowed to air dry and consequently immunoblotted using primary antibodies 6E10 (1:1000, Covance, cat. no. SIG-39320), A11 (1:1000, Millipore, cat. no. AB9234), OC (1:1000, Millipore, cat. no. AB2286) and corresponding HRP conjugated secondary antibodies. Antibody reactivity was visualized using the ECL reagent. Bioluminescence was assessed in a Chemidoc MP imaging system (Bio-Rad, Munich, Germany). Analysis of the Aβ solution by dot blot revealed A11 immunoreactivity only in the brain homogenate of a plaque-containing 5XFAD mouse and no A11 immunoreactivity in the unlabelledor labeled synthetic Aβ preparation. Conversely, OC immunoreactivity was seen only in synthetic Aβ samples, indicating the presence of fibrillary material in the used Aβ solutions (Supplement Fig. 2).

### Immunohistochemistry

For analysis OHSC were fixed in 4%-paraformaldehyde in PBS followed by immunofluorescent staining as described elsewhere in detail^26^,^44^. Antibodies were NeuN (1:1000, Millipore, cat. no. MAB377) to stain neuronal nuclei, Iba1 (1:1000, WAKO chemicals, cat. no. 019-19741) to stain microglia, 4′6-Diamidine-2-phenylindol (DAPI, 1:1000, 1 mg/mL solution, ThermoScientific, cat. no. 62248) to stain nuclei. To stain amyloid beta fragments OHSCs were treated for 30 min with 70% formic acid prior to the usual staining protocol, the antibody used was beta amyloid 1−16 (6E10, 1:500, Covance, cat. no. SIG-39320). Secondary antibodies were labeled fluorescently: donkey anti-mouse-IgG Alexa488 (1:1000, Invitrogen, cat. no. A-21202), donkey anti-rabbit-IgG Alexa647 (1:1000, Invitrogen, cat. no. A-31573). Thioflavin S staining of beta-sheet rich structures was performed according to the modified Thioflavin S staining introduced by Sun and colleagues^45^, but omitting incubation in potassium permanganate since this might interfere with co-immunolabeling. Thioflavin S was purchased from Sigma-Aldrich (cat. no. T-1892), solved in sterile PBS to a stock solution of 0.1% (w/V) and filtered before further dilution. Briefly, OHSCs were incubated for 8 min with 0.002% Thioflavin S in PBS, washed twice for 1 min in 50% ethanol and 5 min in PBS. Afterwards OHSCs were processed for immunofluorescent staining.

### Imaging and image analysis

Immunofluorescently stained OHSC were analyzed by confocal laser scanning microscopy using the ZEISS LSM 510 META. Overview images of whole OHSC were done with a C-Apochromat 10 × /0.45 W objective, high magnification or z-stack images were obtained using a LD LCI Plan-Apochromat 25 × /0.8 Imm. Korr. DIC objective. Thioflavin S staining was quantified in 2D images using the software Image J 1.47v (National Institute of Health) by thresholding the Thioflavin S staining and determining percentage of area covered. 3D single cell and surface reconstruction was performed on z-stacks (0.8 μm image interval) using the software IMARIS 7.6.5 (Bitplane). IMARIS surface creation was done by automatic detection. To view inside the cell, the surface was cut using the clipping plane. The IMARIS plugin filament tracer allows the reconstructions and tracing of microglia filaments. The automatic detection mode was applied for filament creation in a defined region of interest as described before^26^. 10 cells per group were analyzed morphometrically.

### Immunoblotting

Tissue pellets (−80 °C) of hippocampal slice cultures were lyzed in ice-cold lysis buffer (pH 6.8, 42 mM Tris-HCl, 1.3% SDS, 6.5% glycerol, 100 μM sodium orthovanadate in double-destilled water) for 10 minutes, triturated with a pipette, and centrifuged (13,000 × g, 15 min, 4 °C). Next total protein content was measured using a bicinchoninic acid assay (Pierce). Samples (20 μg total protein per lane) were analyzed using Novex® 10–20%-Tricine Protein Gels (Thermo Scientific) and SDS-Page according to manufacturer’s instructions. After antigen retrieval nitrocellulose membranes were blocked for 1 h in Tris-buffered saline containing 0.1% Tween 20 (TBS-T) and 5% nonfat dried milk. Then, the membranes were incubated with mouse anti beta amyloid 1−16 (6E10, 1:500, Covance) in blocking solution overnight, at 4 °C. After washing with TBS-T, the membranes were incubated with horseradish peroxidase-conjugated anti-mouse IgG antibody (1:25,000 in TBS-T containing 1% BSA, GE Healthcare) for 1 hr at room temperature. The immunoreactive bands were visualized using an ECL western blot detection system (GE Healthcare) and analyzed using Fusion-SL image acquisition system (Peqlab, Erlangen, Germany). To confirm equal protein loading, membranes were probed with antibody recognizing actin (1:5,000, Sigma). For semiquantitative analysis four western blots per condition were analyzed for each antibody. Films were digitally scanned and densitometry conducted using Image J 1.40 analysis software. To control for inconsistencies in loading protein bands of interest were normalized to actin loading controls.

### Electron microscopy

#### Fixation

OHSC (at least n = 6 for each condition) were fixed with 4% paraformaldehyde and 0.1% glutaraldehyde in 0.1 M phosphate buffer (PB) for 60 minutes at 4 °C.

#### Immunohistochemistry

For immuno-electron microscopy OHSCs were washed in 50 mM TBS and blocked in 20% normal goat serum (NGS) in TBS for 1 h. Subsequently, the cultures were incubated with primary antibodies rabbit anti-Iba1 (1:200) and mouse anti-6E10 (1:100) diluted in TBS containing 2% NGS overnight at 4 °C. After 1 h of washing in TBS, the OHSCs were incubated with a biotinylated anti-rabbit (for Iba1, 1:100) and a 1.4 nm gold-coupled anti-mouse secondary antibody (for 6E10; 1:100) in TBS containing 2% NGS overnight at 4 °C. OHSCs were rinsed in TBS and then fixed for 10 minutes in a 1% GA solution. Tissue-bound gold particles were enlarged using a silver intensification kit (HQ-Silver, Nanoprobes, USA). Next, sections were washed in double-destilled water for 10 min and 50 mM TB for 1 hr. Visualization of antibody binding by diaminobenzidine (DAB) staining was performed using the ABC Standard Kit (Vector Laboratories, Burlingame, USA) with DAB and H_2_O_2_ as substrates in accordance with the manufacturer’s suggestions. The sections then underwent osmification for 40 min in a solution of 0.5% OsO_4_ and 6.86% sucrose.

#### Embedding

Following osmification, OHSCs were washed in PB followed by 50% and 60% ethanol (EtOH) for 10 minutes each. The tissue was then incubated in 1% uranyl acetate in 70% EtOH for 35 minutes, followed by 10 min dehydration steps in increasing grades of EtOH. After washing in propylene oxide, the tissue was embedded in Durcopan (Fluka). Ultrathin sections (60 nm) of selected hippocampal areas were cut (Leica EM UC6) and mounted on Formvar-coated nickel grids. Sections were viewed and examined in an electron microscope (LEO 906 E, Zeiss, Oberkochen, Germany) and digital images were captured using ISP Software (Tröndle, Germany).

For further characterization of synthetic Aβ samples were loaded on a 300 mesh formvar/carbon coated copper grid (Plano). The samples were fixed for 5 minutes in 1% glutaraldehyde. After washing with double-destilled water the samples were negatively stained for 1 minute with 1% uranyl acetate. As shown in supplementary Fig. 2 fibrils were observed in the used Aβ solutions.

### Statistical analyses

Morphometric and densitometric data were statistically analysed using the SPSS (IBM) software. Data were tested for normal distribution using the Kolmogorov-Smirnov test. If data were normally distributed, 2-sided unpaired Student’s *t*-test was applied. If the data did not meet the criteria of normal distribution the Mann-Whitney *U* test was applied. For multiple comparisons data were analysed using One-way ANOVA followed by Scheffé post-hoc test. Differences were accounted as significant at p < 0.05.

## Additional Information

**How to cite this article**: Hellwig, S. *et al.* Forebrain microglia from wild-type but not adult 5xFAD mice prevent amyloid-b plaque formation in organotypic hippocampal slice cultures. *Sci. Rep.* **5**, 14624; doi: 10.1038/srep14624 (2015).

## Supplementary Material

Supplementary Information
