# Supplementary material for: Forebrain microglia from wild-type but not adult 5xFAD mice prevent amyloid-β plaque formation in organotypic hippocampal slice cultures
Source: Sci Rep. 2015 Sep 29;5:14624. doi: 10.1038/srep14624 (PMC4586757; doi:10.1038/srep14624)

Supplementary Material

Forebrain microglia from wild-type but not adult 5xFAD mice prevent amyloid-β plaque formation in organotypic hippocampal slice cultures

Sabine Hellwig, MD[1*], Annette Masuch, PhD[1*], Sigrun Nestel[2], Natalie Katzmarski[3]

Melanie Meyer-Luehmann, PhD[3], Knut Biber, PhD[1,4,#]

[1]Department of Psychiatry and Psychotherapy, [2]Department of Neuroanatomy, [3]Department of Neurology, University of Freiburg, Freiburg, Germany. [4]Department of Neuroscience, University Medical Center Groningen, University of Groningen, Groningen, The Netherlands.

* These authors contributed equally to the study.

Figure Legends

Supplementary Figure 1 Morphological comparison of replenished microglia isolated from the forebrain of juvenile or young adult wild type or 5xFAD mice. Single cell 3D reconstruction of replenished Iba1-labeled microglia isolated from 5-week- **(A, C)** or 6-month-old **(B, D)** mice. Comparison of morphometric parameters "number of branch points" and "total process length" (**E**, n=10 cells from 3 different OHSC per group) illustrated comparable levels of ramification. Scale bar: 10 µm.

Supplementary Figure 2 Analysis of synthetic amyloid $\beta_{1\text{-}42}$ preparations. (A) Analysis by dot blot revealed A11 immunoreactivity only in the brain homogenate of a plaque-containing 5XFAD mouse and no A11 immunoreactivity in the unlabelled nor labeled synthetic Aβ preparation. Conversely, OC immunoreactivity was seen in synthetic Aβ preparations, indicating the presence of fibrillary material in the used Aβ solutions. **(B)**These findings were confirmed by EM experiments were several fibrils were seen in FAM-labeled $A\beta_{1\text{-}42}$ solution**.** Asterisks mark single fibrils. Scale bar: 250 nm.

Supplementary Figure 1

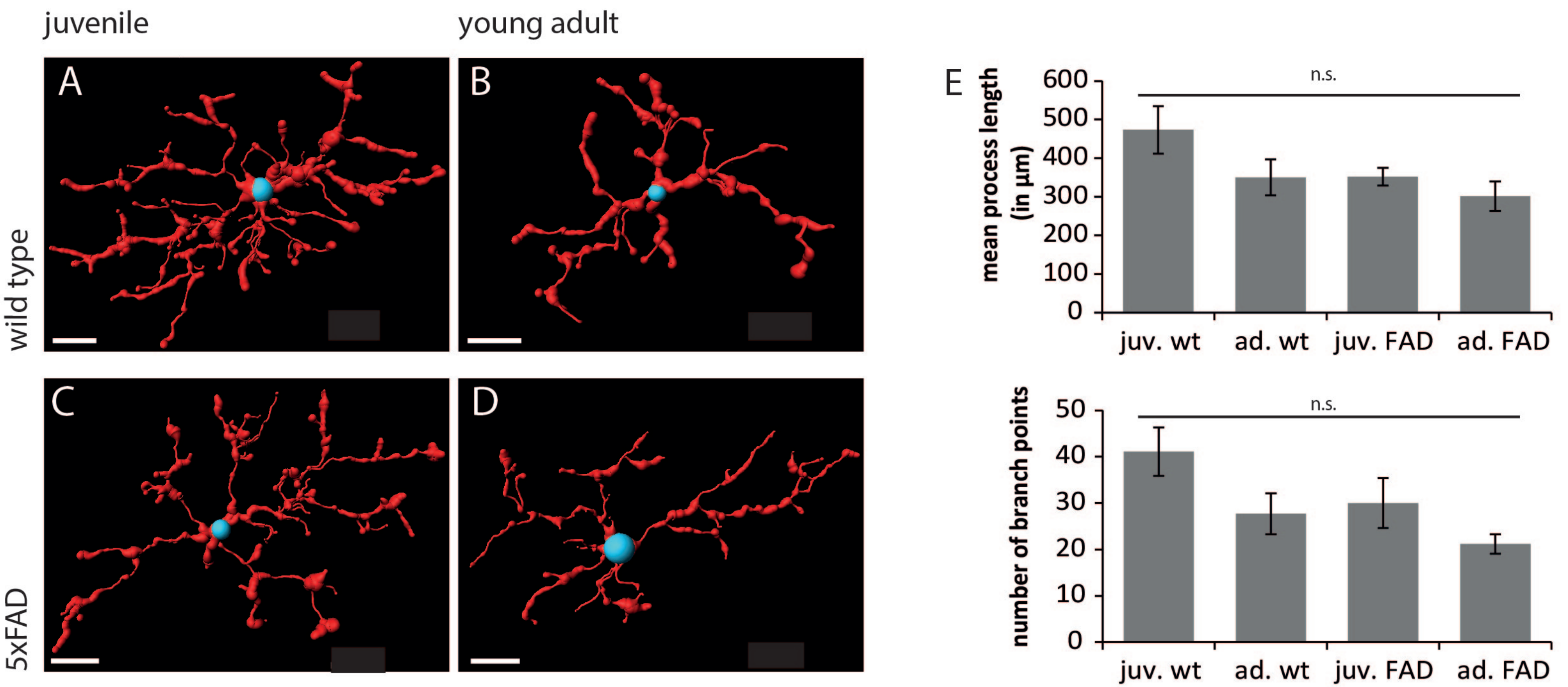

Supplementary Figure 2

A

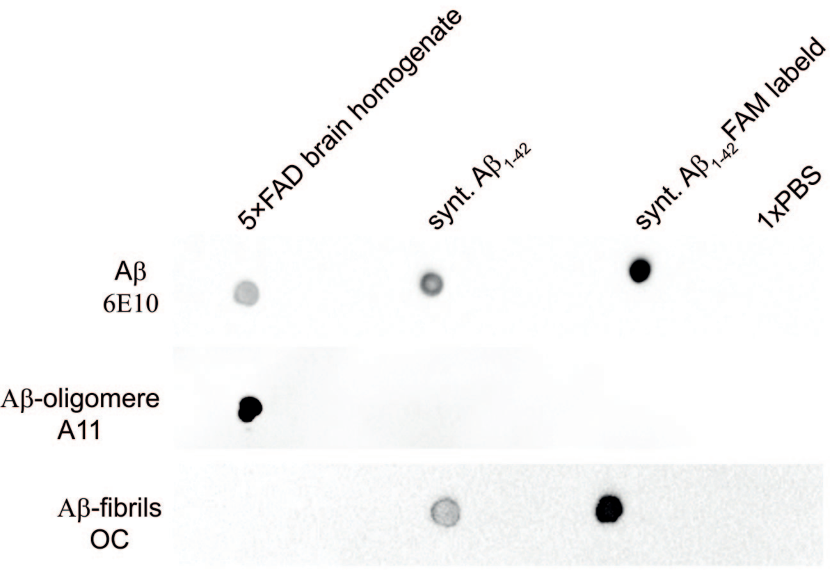

B

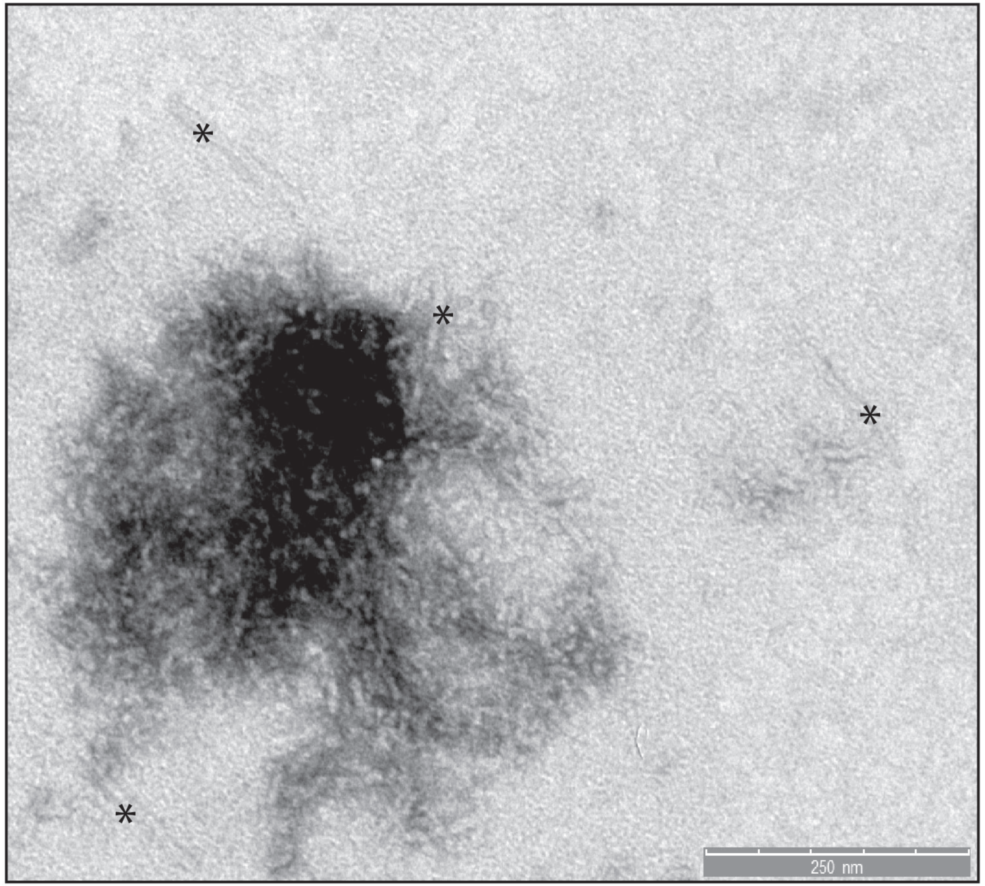

Supplement: Supplementary Information [file srep14624-s1.pdf]
